# Supplementary material for: Splice-Junction-Based Mapping of Alternative Isoforms in the Human Proteome
Source: Cell Rep. Author manuscript; Available in PMC 2020 Jan 15. (PMC6961840; doi:10.1016/j.celrep.2019.11.026)
Supplement: 2 [file NIHMS1546469-supplement-2.zip › DF1/PXD006675/CardiacFibroblast/CardiacFibroblast_21_TOGARAM1_NLVQQDFLLQR.pdf]

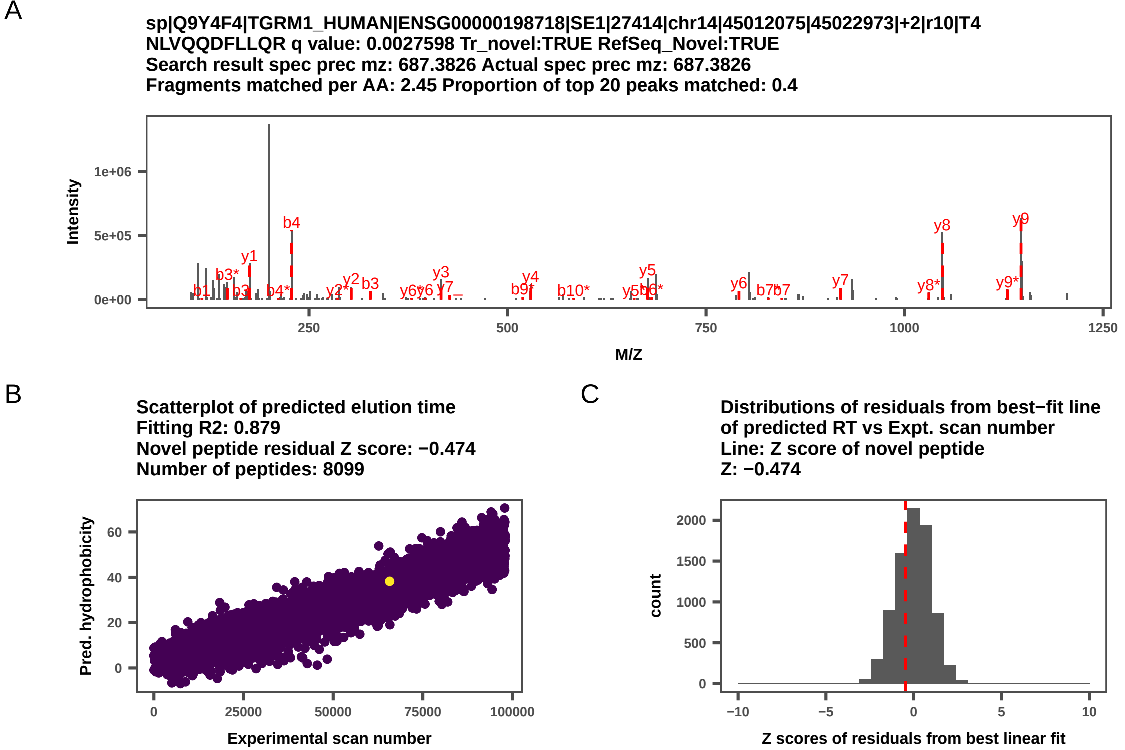
A
sp|Q9Y4F4|TGRM1_HUMAN|ENSG00000198718|SE1|27414|chr14|45012075|45022973|+2|r10|T4
NLVQQDFLLQR q value: 0.0027598 Tr_novel:TRUE RefSeq_Novel:TRUE
Search result spec prec mz: 687.3826 Actual spec prec mz: 687.3826
Fragments matched per AA: 2.45 Proportion of top 20 peaks matched: 0.4
Intensity
1e+06
5e+05
0e+00
b4
y1
b3*
b1
b3
b4*
y2
y2*
b3
y3
y6*
y6
y7
y4
b9*
b10*
y5
y5*
b6*
y6
b7*
b7
y7
y8
y8*
y9
y9*
250
500
750
1000
1250
M/Z
B
Scatterplot of predicted elution time
Fitting R2: 0.879
Novel peptide residual Z score: −0.474
Number of peptides: 8099
Pred. hydrophobicity
60
40
20
0
0
25000
50000
75000
100000
Experimental scan number
C
Distributions of residuals from best−fit line
of predicted RT vs Expt. scan number
Line: Z score of novel peptide
Z: −0.474
count
2000
1500
1000
500
0
−10
−5
0
5
10
Z scores of residuals from best linear fit
